# To split or not to split? Multilocus phylogeny and molecular species delimitation of southeast Asian toads (family: Bufonidae)

**DOI:** 10.1186/s12862-019-1422-3

**Published:** 2019-04-25

**Authors:** Kin Onn Chan, L. Lee Grismer

**Affiliations:** 1 Lee Kong Chian Natural History Museum, National University of Singapore, 2 Conservatory Drive, Singapore, 117377 Singapore; 2 Herpetology Laboratory, Department of Biology, La Sierra University, 4500 Riverwalk Parkway, Riverside, CA 92515 USA

**Keywords:** BPP, Genealogical divergence index, Gdi, Systematics, Species boundaries

## Abstract

**Background:**

Recent studies have demonstrated that Bayesian species delimitation based on the multispecies coalescent model can produce inaccurate results by misinterpreting population splits as species divergences. An approach based on the genealogical divergence index (*gdi*) was shown to be a viable alternative, especially for delimiting allopatric populations where gene flow is low. We implemented these analyses to assess species boundaries in Southeast Asian toads, a group that is understudied and characterized by numerous unresolved species complexes.

**Results:**

Multilocus phylogenetic analyses showed that deep evolutionary relationships including the genera *Sigalegalephrynus, Ghatophryne, Parapelophryne, Leptophryne, Pseudobufo, Rentapia,* and *Phrynoides* remain unresolved. Comparison of genetic divergences revealed that intraspecific divergences among allopatric populations of *Pelophyrne signata* (Borneo vs. Peninsular Malaysia), *Ingerophrynus parvus* (Peninsular Malaysia vs. Myanmar), and *Leptophryne borbonica* (Peninsular Malaysia, Java, Borneo, and Sumatra) are consistent with interspecific divergences of other Southeast Asian bufonid taxa. Conversely, interspecific divergences between *Pelophryne guentheri/P. api, Ansonia latiffi/A. leptopus,* and *I. gollum/I. divergens* were low (< 3%) and consistent with intraspecific divergences of other closely related taxa. The BPP analysis produced variable results depending on prior settings and priors estimated from empirical data produced the best results that were also congruent with the *gdi* analysis.

**Conclusions:**

This study showed that the evolutionary history of Southeast Asian toads is difficult to resolve and numerous relationships remain ambiguous. Although some results from the species delimitation analyses were inconclusive, they were nevertheless efficacious at identifying potential new species and taxonomic incompatibilities for future in-depth investigation. We also demonstrated the sensitivity of BPP to different priors and that careful selection priors based on empirical data can greatly improve the analysis. Finally, the *gdi* can be a robust tool to complement other species delimitation methods.

**Electronic supplementary material:**

The online version of this article (10.1186/s12862-019-1422-3) contains supplementary material, which is available to authorized users.

## Background

The instability of Southeast Asian amphibian systematics cannot be overstated and reflects the rate at which new insights are gained, largely through the use of molecular data [1]. The increased effort to sequence rare, historical, and widespread taxa at a finer genetic and geographical scale has brought upon a renewed understanding of the region’s complex biodiversity patterns and evolutionary history. Consequently, taxonomic nomenclature is constantly in flux [2–11], as it attempts to keep pace with the increasing frequency at which new species are being described (especially cryptic species) and revised [12–26]. Although these insights have improved our understanding of Southeast Asian amphibian biodiversity considerably, many taxa have yet to be sequenced or subjected to molecular analyses, and more fine-scale geographic sampling is still needed to adequately characterize species boundaries and distribution ranges.

True toads (family Bufonidae) have a native distribution throughout Asia, Africa, Europe, and America, but have also been widely introduced to Australia and the Oceanic islands [27]. Southeast Asian taxa represent a relatively small portion of the family’s diversity and consequently, have received comparatively less research attention. However, studies have begun to show that the diversity of Southeast Asian toads is severely underestimated and much of their evolutionary history remains unresolved [13, 20, 21, 26, 28–30]. Moreover, most research focused on small subsets relating to specific taxon groups, thereby precluding the detection of broad biodiversity and evolutionary patterns that can only be revealed by more comprehensive studies conducted at a broader scale [5].

Although many Southeast Asian toad species have been sequenced and are publicly available on GenBank, these sequences have not been collated and analysed within a comprehensive phylogenetic framework. This includes widespread taxa that are co-distributed across separate landmasses and distinct biogeographic regions such as Borneo, Sumatra, Java, Peninsular Malaysia, and Indochina. Such taxa are of particular interest because they are phenotypically similar, yet have not been analysed within a broader phylogeographic context. Studies that have included wider geographic sampling have revealed that widespread species were composed of multiple undescribed lineages with more fragmented distributions [7, 16, 21, 31–39]. However, no such study has been conducted on Southeast Asian toads, thereby highlighting the urgent need for more in-depth studies to fill the obvious gap in our understanding of the group’s evolution, biodiversity, and distribution. This study aims to provide a comprehensive working phylogeny and preliminary analysis of genetic variation, phylogeographic structure, and species boundaries, with emphasis on cryptic complexes that are co-distributed across multiple biogeographic regions.

The use of molecular data and development of statistical species delimitation methods have become indispensable tools to aid in the detection and delimitation of new species [40, 41]. Among these, methods based on the multispecies coalescent (MSC) model are largely regarded as robust, especially for closely related species. However, studies based on simulations and empirical data have revealed that certain MSC-based methods may over-split by delimiting populations as species [22, 42, 43]. Widely used species delimitation programs such as Bayesian Phylogenetics and Phylogeograpy (BPP; Yang and Rannala, 2010) were found to be unable to differentiate population structure from species cladogenesis when sequence data were simulated under the protracted speciation model [42]. Subsequently, the empirical genealogical divergence index (*gdi*) was proposed and found to be more reliable than BPP in differentiating population structure from species divergence, especially for allopatric populations [43, 44]. As such, the main goals of this study are to: 1) estimate a comprehensive multilocus phylogeny of Southeast Asian bufonids to elucidate ambiguous evolutionary relationships; and 2) implement a multi-step approach to species discovery and delimitation to screen for potential new species and taxonomic incompatibilities. We first applied mitochondrial divergence thresholds specific to amphibians to identify candidate species for species delimitation analyses [45, 46]. We then used BPP to test putative species boundaries [47] and the *gdi* analysis to validate the results [43, 44].

## Results

### Phylogenetic estimation

**Fig. 1:**
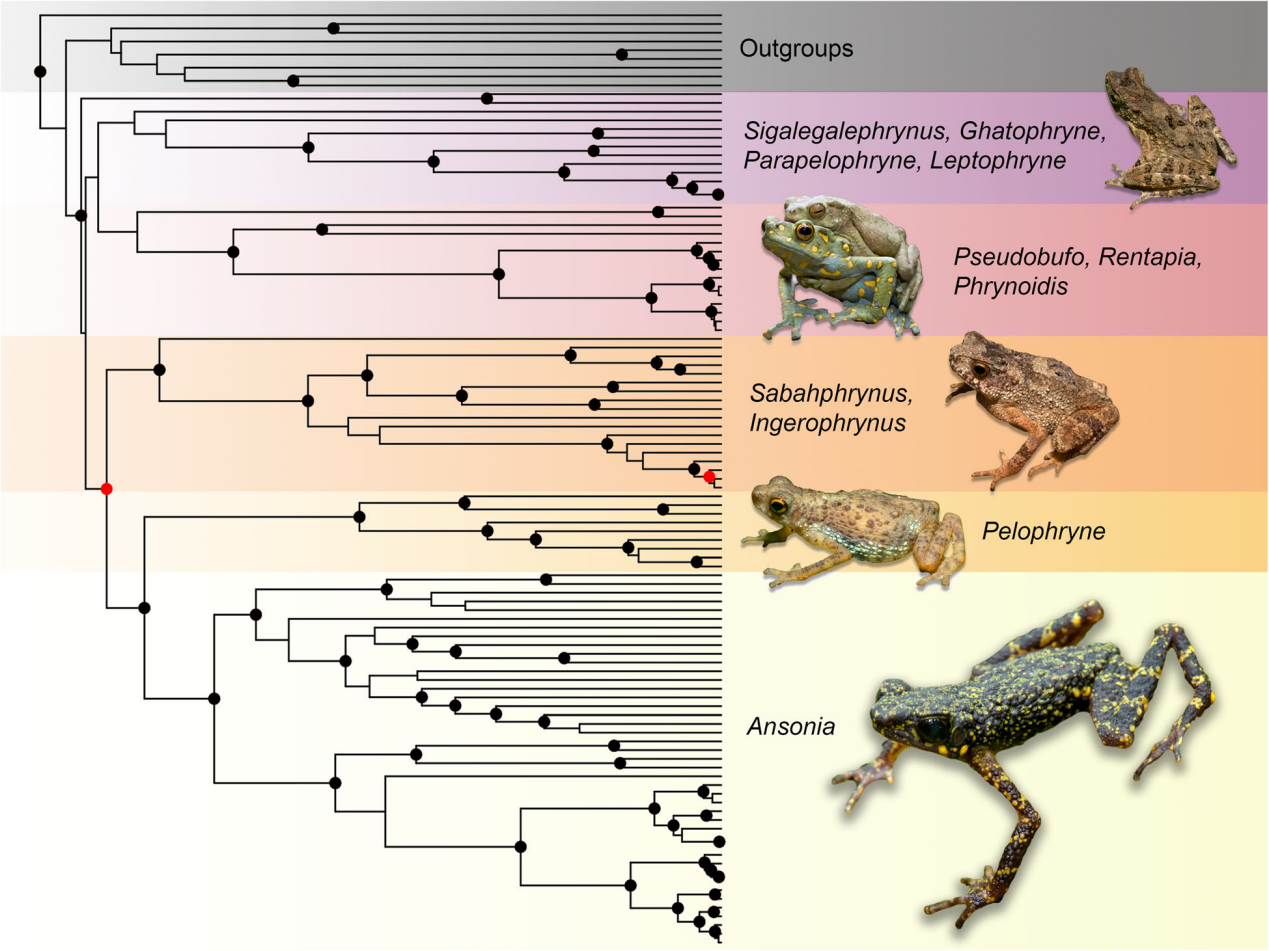
Bayesian phylogeny of derived from 6236 bp comprising three mitochondrial (12S, 16S, CO1) and three nuclear genes (CXCR4, NCX1, RAG-1). Black dots denote branch support with PP ≥0.95, while red dots denote 0.9 ≤ PP < 0.95. All images in the figure were taken by the first author

The best-fit substitution model scheme for the ML analysis was GTR + F + R5 for the partitions 12S and 16S; TIM2 + F + I + G4 for the partition CO1; and TN + F + G4 for the partitions CXCR4, NCX1, and RAG-1. Both ML and BEAST phylogenies produced largely congruent results. At the generic level, the ML tree was poorly supported at the basal nodes, including the genera *Sigalegalephrynus, Parapelophryne, Leptophryne, Pseudobufo, Ghatophryne, Rentapia,* and *Phrynoidis*. Relationships of the other genera (*Sabahphrynus, Ingerophrynus, Pelophryne,* and *Ansonia*) were relatively well resolved. The BEAST tree differed only in the placement of the genus *Ghatophryne*, which was recovered as the sister lineage to *Parapelophryne* + *Leptophryne*, as opposed to the ML tree, where it was the sister lineage to *Rentapia* + *Phrynoidis*. Species-level relationships were also largely congruent, with a few exceptions including the placement of *Ansonia kraensis*, *A. inthanon* and *I. gollum*. The phylogenetic placement of *Ingerophrynus gollum* and its closest relative, *I. divergens* was not well-resolved in both Bayesian and ML phylogenies. In the Bayesian phylogeny, *I. divergens* was paraphyletic with respect to *I. gollum*, however, the clade lacks statistical support (Additional file 2). In the ML phylogeny, *I. gollum* and *I. divergens* were recovered as sister species but the entire clade lacked phylogenetic structure and support (Additional file 1). Branch support was relatively high for both phylogenies except for the most basal splits. While the ML phylogeny recovered a polytomy at the base of the tree, the BEAST phylogeny recovered the genus *Sigalegalephrynus* as the earliest diverging ingroup with high support (PP = 1.0; Fig. 1, Additional files 1, 2).

### Identification of candidate species

**Fig. 2:**
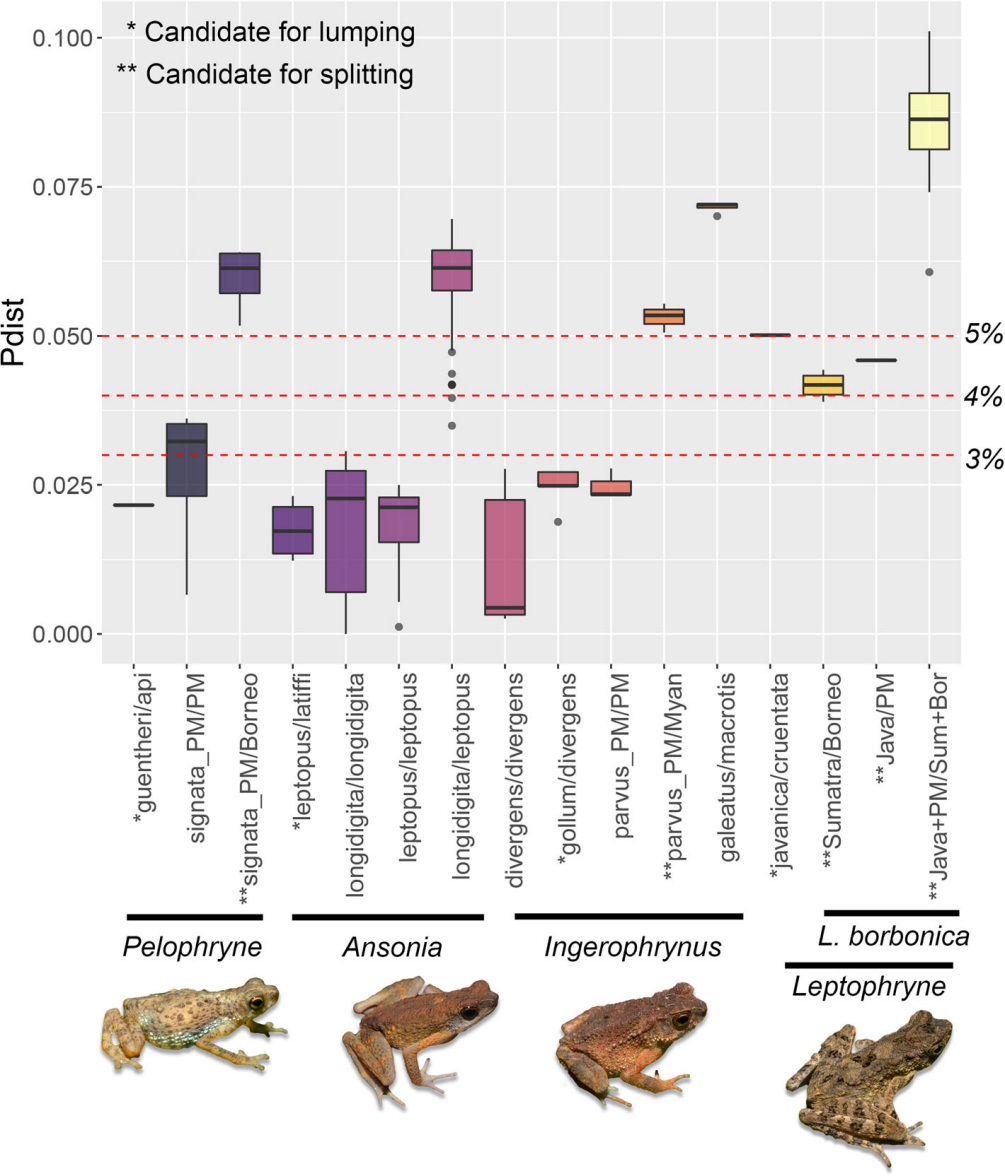
Threshold-based approach to identify candidate species for lumping and splitting using uncorrected p-distances at the 16S gene. Dotted red lines show divergence thresholds of 3–5%. All images in the figure were taken by the first author

Below the 3% threshold, three taxon pairs were identified as candidates for lumping (Fig. 2). These include *Pelophryne guentheri/P. api* (2.2%), *Ansonia leptopus/A. latiffi* (1.2–2.3%), and *Ingerophrynus gollum/I. divergens* (1.9–2.7%). Intraspecific divergences among populations of *Pelophryne signata* from Peninsular Malaysia exceeded this threshold (0.7–3.6%). From 4 to 5%, another taxon pair, *Leptophryne cruentata/L. javanica* (5%) was identified for lumping, whereas two other taxon pairs were identified for splitting. These include, populations of *Leptophryne borbonica* from Borneo vs. Sumatra and from Java vs. Peninsular Malaysia (PM). Above the 5% threshold, three taxa were earmarked for splitting including *Pelophryne signata* from Borneo vs. PM (5.2–6.4%), *Ingerophrynus parvus* from PM vs. Myanmar (5.1–5.5%), and *L. borbonica* from Java+PM vs. Sumatra+Borneo (6.1–10.1%; Fig. 2). Individual pairwise distances for all populations are presented in Additional file 5.

### Species delimitation

**Fig. 3:**
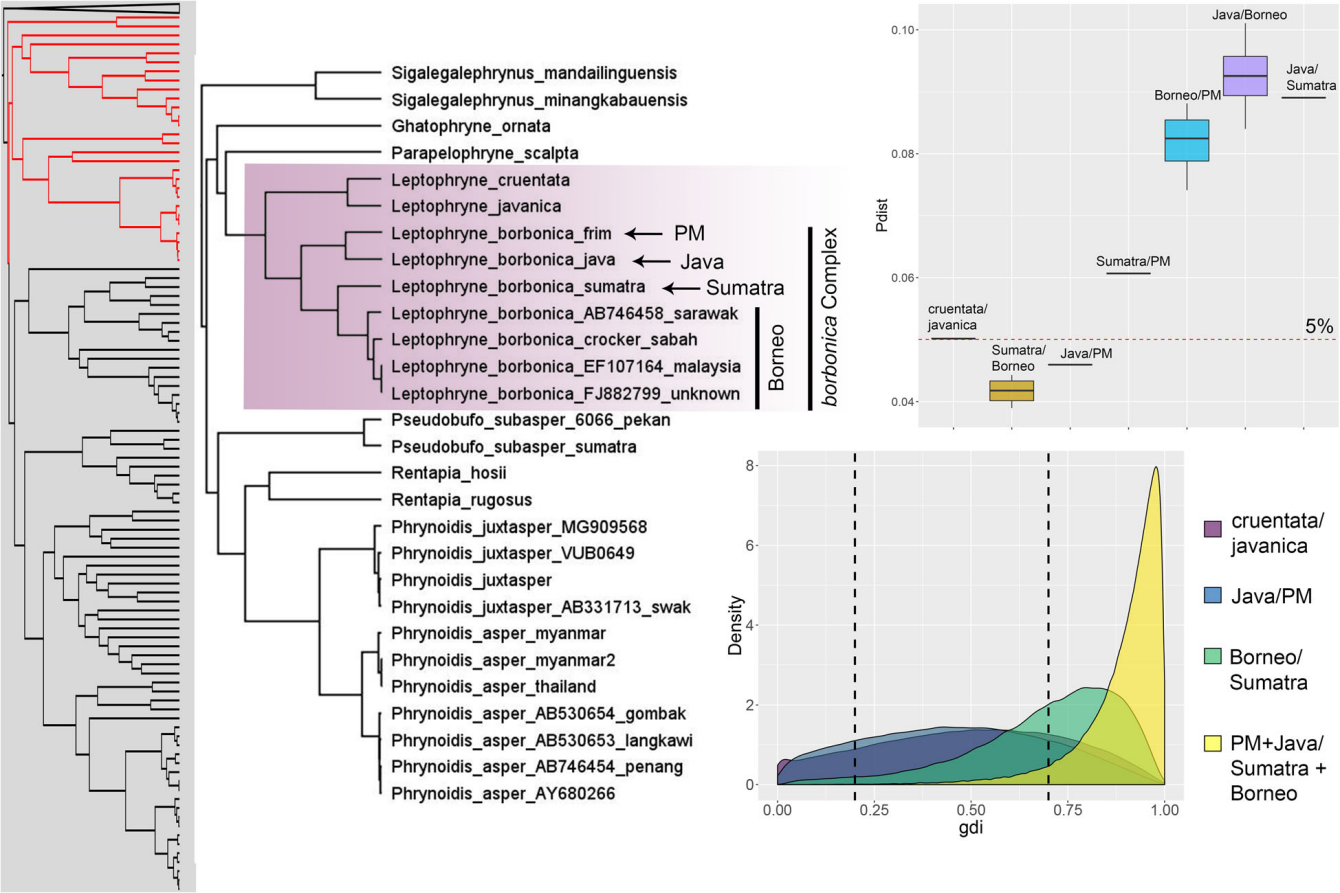
Left: subset of the Bayesian phylogeny showing the genera *Sigalegalephrynus, Ghatophryne, Parapelophryne, Leptophryne, Pseudobufo, Rentapia*, and *Phrynoidis*. Highlighted tips are candidate taxa included in species delimitation analyses. Top right: boxplots of uncorrected p-distances (16S) for the genus *Leptophryne*. Bottom right: density plot of *gdi* values. Populations are considered distinct species when *gdi* values are > 0.7, while low *gdi* values < 0.2 indicate that populations belong to the same species. Values of 0.2 > *gdi* < 0.7 indicate ambiguous species status

**Fig. 4:**
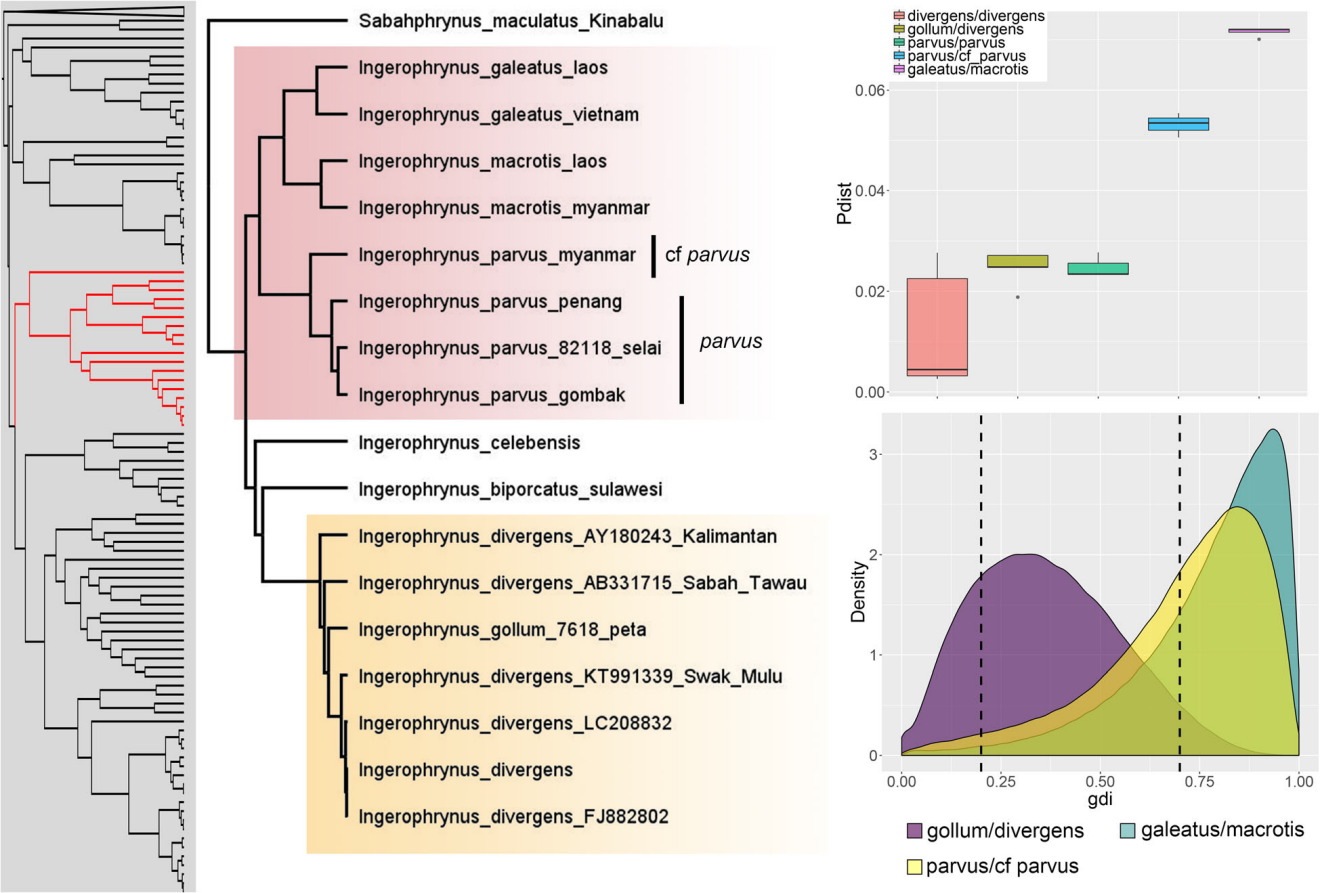
Left: subset of the Bayesian phylogeny showing the genera *Sabahphrynus* and *Ingerophrynus*. Highlighted tips are candidate taxa included in species delimitation analyses. Top right: boxplots of uncorrected p-distances (16S) for the genus *Ingerophrynus*. Bottom right: density plot of *gdi* values

A comparison between posterior distributions generated from the priors and empirical data showed that there was sufficient information in the data and that results were not driven by the priors (Additional file 3). Overall, the A10 and A11 analyses produced similar results, indicating that the estimated species tree did not differ from the inferred Bayesian phylogeny. The exception was the comparison between *Ingerophrynus gollum/divergens* where posterior probabilities for the A10 and A11 analyses using empirical priors were markedly different (0.58 vs. 0.86). This was not unexpected considering the uncertain phylogenetic placement of this species (Additional files 1 and 2). Both A10 and A11 analyses were sensitive to prior settings and posterior probabilities based on empirical priors were generally lower than those based on pre-defined priors (Tables 1 and 2). For the candidates for lumping, certain non-empirical prior sets were highly supported but support was weak when empirical priors were employed (Table 1). This was corroborated by the uncertain values in the *gdi* analysis (Table 3). For the candidates for splitting, BPP analyses based on empirical priors and the *gdi* highly supported the distinction between *Leptophryne borbonica* from Java + PM/Sumatra + Borneo and *Pelophryne signata/*cf. *signata* as separate species (Tables 2 and 3). The *gdi* analysis also supported the split between *L. borbonica* from Sumatra vs. Borneo (mean ± SD = 0.70 ± 0.19; Fig. 3) and between *Ingerophrynus parvus* from PM vs. Myanmar (mean ± SD = 0.71 ± 0.20; Fig. 4). However, these were weakly and moderately supported in the BPP analyses (pp = 0.68 and 0.87 respectively). Complete results for every A11 analyses performed are provided in Additional file 6.

**Table 1:** Posterior probabilities from both BPP analyses (A10 | A11) for the candidates for lumping under different prior settings. A10 = species delimitation on a guide tree; A11 = joint species delimitation and species tree estimation

		Candidates for lumping			
Prior (θ)	Prior (τ)	cruentata/ javanica	gollum/ divergens	api/ guentheri	latiffi/ leptopus
IG(3, 0.0002)	IG(3, 0.004)	1.0 | 1.0	0.49 | 0.97	1.0 | 1.0	0.00 | 0.22
IG(3, 0.002)	IG(3, 0.004)	1.0 | 0.98	0.97 | 0.98	1.0 | 1.0	1.0 | 1.0
IG(3, 0.02)	IG(3, 0.004)	0.97 | 0.83	0.90 | 0.84	0.52 | 0.64	0.99 | 1.0
IG(21, 0.02)	IG(3, 0.004)	1.0 | 0.94	0.99 | 0.94	1.0 | 1.0	1.0 | 1.0
IG(21, 0.2)	IG(3, 0.004)	0.94 | 0.87	0.99 | 0.88	0.50 | 0.62	0.96 | 0.96
IG(3, 0.002)	IG(3, 0.0004)	0.62 | 0.80	0.80 | 0.80	0.54 | 0.71	0.98 | 0.99
IG(3, 0.002)	IG(3, 0.04)	1.0 | 1.0	1.0 | 1.0	1.0 | 1.0	1.0 | 1.0
IG(3, 0.002)	IG(21, 0.004)	0.68 | 0.80	0.90 | 0.80	0.65 | 0.71	0.98 | 0.99
IG(3, 0.002)	IG(21, 0.04)	0.95 | 0.99	0.99 | 0.99	0.96 | 0.97	1.0 | 1.0
IG(3, 0.002)	IG(21, 0.4)	1.0 | 1.0	1.0 | 1.0	1.0 | 1.0	1.0 | 1.0
Empirical	Empirical	0.46 |0.47	0.58 | 0.86	0.39 | 0.46	0.85 | 0.87

**Table 2:** Posterior probabilities from both BPP analyses (A10 | A11) for the candidates for splitting under different prior settings. A10 = species delimitation on a guide tree; A11 = joint species delimitation and species tree estimation

		Candidates for splitting				
Prior (θ)	Prior (τ)	borbonica Java/PM	borbonica Sumatra/ Borneo	borbonica Java+PM/ Sum+Bor	parvus PM/Myanmar	signata/cf. signata
IG(3, 0.0002)	IG(3, 0.004)	1.0 | 0.93	0.86 | 0.92	0.86 | 1.0	0.62 | 0.48	0.93 | 0.0
IG(3, 0.002)	IG(3, 0.004)	0.88 | 1.0	0.99 | 1.0	0.99| 0.95	0.93 | 0.94	1.0 | 0.96
IG(3, 0.02)	IG(3, 0.004)	0.85 | 0.83	0.95| 0.83	1.0 | 0.82	0.84 | 0.79	0.78 | 0.89
IG(21, 0.02)	IG(3, 0.004)	1.0 | 1.0	1.0 | 1.0	1.0 | 1.0	0.97 | 0.95	1.0 | 1.0
IG(21, 0.2)	IG(3, 0.004)	0.94 | 0.95	1.0 | 0.98	1.0 | 1.0	0.99 | 0.98	0.77 | 0.96
IG(3, 0.002)	IG(3, 0.0004)	0.70 | 0.80	0.86 | 0.79	0.97 | 0.85	0.81 | 0.84	0.77 | 0.70
IG(3, 0.002)	IG(3, 0.04)	1.0 | 1.0	1.0 | 1.0	1.0 | 1.0	1.0 | 1.0	1.0 | 1.0
IG(3, 0.002)	IG(21, 0.004)	0.71 | 0.81	0.91 | 0.82	0.99 | 0.9	0.87 | 0.87	0.82 | 0.75
IG(3, 0.002)	IG(21, 0.04)	0.96 | 0.97	0.98 | 0.97	0.99 | 0.98	0.78 | 0.96	0.89 | 0.87
IG(3, 0.002)	IG(21, 0.4)	1.0 | 1.0	1.0 | 1.0	1.0 | 1.0	1.0 | 1.0	1.0 | 1.0
Empirical	Empirical	0.50 | 0.55	0.67 | 0.68	1.0 | 0.99	0.86 | 0.87	0.97 | 0.98

**Table 3:** Summary of the different lines of evidence used for species delimitation. BPP support summarizes results from A10 and A11 analyses using empirical priors. PP ≥ 0.95 denotes high support, 0.90 ≤ pp. < 0.95 denotes moderate support, and pp. < 0.9 denotes weak support

Candidates for lumping	Mitochondrial divergence	BPP support	*gdi* (mean ± SD)
*Leptophryne cruentata/L. javanica*	5.00%	Weak	0.49 ± 0.25
*Ingerophrynus gollum/I. divergens*	1.9–2.7%	Weak	0.37 ± 0.18
*Pelophryne api/P. guentheri*	2.20%	Weak	0.38 ± 0.23
*Ansonia latiffi/A. leptopus*	1.2–2.3%	Moderate	0.62 ± 0.21
Candidates for splitting			
*Leptophryne borbonica:*			
Java | PM	4.60%	Weak	0.46 ± 0.23
Sumatra | Borneo	3.9–4.4%	Weak	0.70 ± 0.19
Java + PM | Sumatra + Borneo	6.1–10.1%	High	0.89 ± 0.11
*Ingerophrynus parvus* (PM vs. Myanmar)	5.1–5.5%	Moderate	0.71 ± 0.20
*Pelophryne signata* (PM + Sumatra vs. Borneo)	5.2–6.4%	High	0.76 ± 0.18

## Discussion

### Evolutionary relationships

The phylogenetic relationships inferred from this study bear similarities as well as disagreements with previous studies. The most recent and comprehensive phylogenetic study on the family Bufonidae focused on Arabian taxa but included numerous Southeast Asian species [48]. However, the study lacked a number of key genera such as *Pseudobufo*, *Sigalegalephrynus,* and *Parapelophryne* that were unavailable from GenBank at that point in time. Similar to our results, the backbone of their tree was mostly unresolved and poorly supported. Their phylogeny differed in a number of major relationships, including the placement of the *Rentapia* + *Phrynoidis* clade, which they recovered as sister to the *Pelophryne* + *Ansonia* clade. Additionally, the genus *Sabahphrynus* (endemic to Borneo) was recovered as the sister lineage of *Strauchbufo raddei* from north and east Asia [27], albeit with low support. Our results inferred *Sabahphrynus* as the sister lineage to the Southeast Asian genus *Ingerophrynus*, which we favour as a more biogeographically plausible scenario that is also strongly supported in both our phylogenies. The morphological similarities between *Sabahphrynus* and other Southeast Asian taxa and its obvious disparity with *Strauchbufo* and other Eurasian taxa also supports this hypothesis. Another relevant multilocus study that focused on the description of *Sigalegalephrynus* from Indonesia [13] included most Southeast Asian genera, but the relationships of major clades were poorly supported and differed drastically from other studies [48, 49], including ours. Overall, our phylogeny bears the most congruence to the phylogeny of Pyron and Wiens [49] with regards to the placements *of Phrynoidis, Rentapia, Sabahphrynus, Ingerophrynus, Ansonia*, and *Pelophryne*. Their phylogeny however, recovered the Southeast Asian genus *Leptophryne* as the sister lineage to the European genus *Epidalea*, which we consider to be less parsimonious from a biogeographic perspective.

What all Southeast Asian toad phylogenies share in common, is the poor support along the backbone of the tree, possibly due to the lack of informative characters/genes or evolutionary processes such as hybridization, rapid radiation and incomplete lineage sorting that can obfuscate phylogenetic signal [50–54]. Even though the most robust phylogenies were estimated using 12 loci [48, 49], basal nodes remained poorly supported, especially with regard to a number of enigmatic genera such as *Pseudobufo*, *Ghatophryne*, *Parapelophryne*, and *Sigalegalephrynus*. These taxa are crucial to the understanding of Southeast Asian biodiversity as they occur in the fringes of the region and represent highly specialized, endemic, and possibly relictual lineages. Moreover, these taxa are represented by relatively few specimens and even fewer molecular samples. The inadequacies of current data to resolve the evolutionary history of Southeast Asian toads highlight the urgent need for more intensive collection efforts and high throughput sequencing at the genomic level to bolster exiting data.

### Reconciling results: limitations and recommendations

Results from our study demonstrate that cryptic species delimitation can be a non-trivial task, especially for taxa in the grey zone of the speciation continuum (0.5–2% molecular divergence) [55]. In some cases, morphological differences can evolve faster than the formation of a species barrier (depressed hybrid fitness), but in others, morphological differentiation may not accompany genetic divergence [56]. This can lead to taxonomic discrepancies and incongruent species delimitation conclusions, especially if different operational criteria are used for species diagnosis.

The decision to split or lump can be straightforward in cases where multiple independent lines of evidence are in agreement. However, different methods can produce conflicting results that are often difficult to interpret and reconcile. The BPP analysis was sensitive to certain prior settings, especially the age of the root (τ_0_). Our results showed that increasing τ to larger values such as 0.04 or 0.4 produced in our opinion, spuriously high posterior probabilities (pp = 1.0 for all tested candidate taxa), possibly because large values of τ pushes coalescent events closer to the tips. The effects of changing θ were less obvious. Larger θ increased posterior probabilities in some cases but not others, indicating that population size and mutation rate need to be estimated on a case-by-case basis depending on the taxa. This demonstrates the sensitivity and potentially misleading results that can be produced by BPP when unrealistic priors are used. Results were more reasonable and congruent with the *gdi* analysis when empirical priors were employed. We therefore caution against using a range of priors that may not accurately represent the diversification and demographic history of the taxa in question, but instead, employ empirical priors that are estimated from the data.

### Candidates for lumping

*Leptophryne cruentata* and *L. javanica* were relatively well-diverged (5%) but were not highly supported as separate species. However, both these taxa were represented by singletons and this was reflected in the *gdi* analysis that produced a flat and diffused posterior distribution. A similar result was seen in *Pelophryne api*/*P. guentheri,* which were also represented by singletons. These results should therefore be interpreted with caution and demonstrates the potential impact of sampling density on species delimitation analyses. For *Ingerophrynus gollum* and *I. divergens*, result of the BPP A10 analysis based on empirical priors was weak (0.6) whereas the A11 analysis was moderate (0.9). The *gdi* analysis was largely uncertain while the mitochondrial divergence was relatively low (2–3%). This, coupled with the relatively short branch lengths, suggests that these lineages were recently separated on their individual landmasses (PM and Borneo), likely during the Pleistocene glaciations and may still be in the early stages of speciation. Surprisingly, although the genetic divergence between *Ansonia latiffi* and *A. leptopus* was low (1–2%), the BPP and *gdi* analysis provided moderately strong support in favour of their recognition as distinct species. This could be due to the significant genetic substructuring and uncertain phylogenetic relationships among the different geographic populations, indicating that the *A. leptopus* group could potentially represent a species complex that requires further investigation. In general, our results did not unequivocally support any of the candidates for lumping, indicating that these taxa could potentially be conspecific or that these lineages are in the nascent stages or grey zone of speciation due to their relatively recent divergence.

### Candidates for splitting

**Fig. 5:**
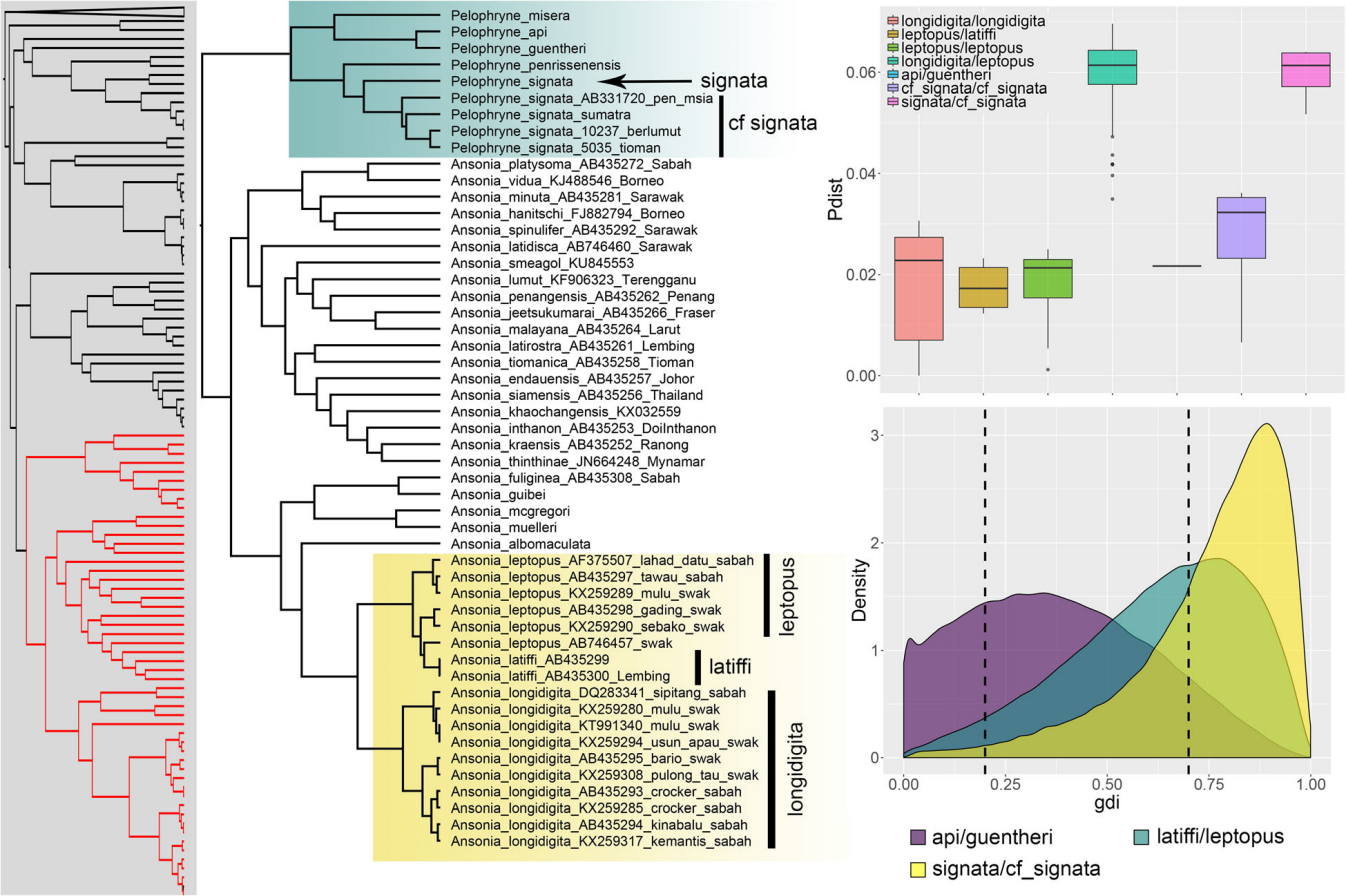
Left: subset of the Bayesian phylogeny showing the genera *Pelophryne* and *Ansonia*. Highlighted tips are candidate taxa included in species delimitation analyses. Top right: boxplots of uncorrected p-distances (16S) for the candidate taxa. Bottom right: density plot of *gdi* values

The genetic divergences among populations of the *Leptophryne borbonica* complex range from 3.9–10.1%, which are clearly within the range of interspecific divergences of other bufonid and amphibian taxa [21, 45, 57, 58]. Based on the type locality, we consider the population from Java to be the true *L. borbonica*, which is the sister lineage to the population from PM. Although the genetic divergence between these two populations was relatively high (4.6%), their recognition as distinct species was not strongly supported by the BPP and *gdi* analyses. However, both populations were represented by singletons and thus, more sampling will be required before stronger conclusions can be made. The genetic divergence between populations from either Sumatra or Borneo with the true *L. borbonica* from Java is high (9–10%; Fig. 3), clearly indicating that they could represent distinct species. When Sumatra + Borneo and Java + PM were compared, all analyses strongly supported those lineages as distinct species. These results show that populations from Sumatra and Borneo are distinct from the true *L. borbonica* from Java and warrants specific recognition. However, it remains unclear whether populations from Sumatra and Borneo, or Java and PM should be lumped as the results showed that they could potentially be distinct from each other as well. Similarly, the genetic divergence between the true *Pelophryne signata* from Borneo and its sister lineage from PM and Sumatra (cf signata) is 5.2–6.4%, which is consistent with interspecific divergences of other closely related taxa (Fig. 5). Additionally, the BPP and *gdi* analyses highly supported the recognition of these populations as distinct species.

The type locality of *Ingerophrynus parvus* is in PM, but has a wide distribution ranging from southern Myanmar, parts of Indochina, along the Malay Peninsula, Sumatra, and western Java [27]. Unfortunately, only samples from Myanmar and PM were available for analysis. Our results showed that these populations are relatively well-diverged (~ 5%), received moderate support in BPP analyses, and high support in the *gdi* analysis for being distinct species. Denser population-level sampling throughout its wide distribution range will likely reveal this group to be a species complex with considerable genetic and phylogeographic structure.

## Conclusions

This study showed that the evolutionary history of Southeast Asian toads is difficult to resolve using traditional Sanger-based loci and that numerous taxon groups require further revision. Although some results from the species delimitation analyses were inconclusive, they were nevertheless efficacious at identifying numerous potential new species and taxonomic incompatibilities for future in-depth investigation. We hold formal taxonomic changes in abeyance, pending the examination of type material and more robust analyses that incorporates more genetic loci and the consideration of additional, independent lines of evidence.

## Methods

### Taxon sampling

We obtained DNA sequence data from 107 ingroup samples including all nine Southeast Asian genera (*Ansonia, Ingerophrynus, Leptophryne, Pelophryne, Phrynoidis, Pseudobufo, Rentapia, Sabahphrynus, Sigalegalephrynus*), representing approximately 50 of the 68 species known to occur in Southeast Asia [27]. Sequences for three mitochondrial (12S, 16S, CO1) and three nuclear genes (CXCR4, NCX1, RAG-1) were acquired from GenBank, including six additional newly sequenced samples from Malaysia (Additional file 4). New sequences were obtained from tissue samples loaned from the La Sierra University Herpetological Collection, Riverside, California, USA and no animals/specimens were used in this study. The six genetic markers were selected because they had the highest coverage across the ingroup taxa. DNA for the new samples was extracted with the Promega Maxwell© RSC Instrument using the Maxwell© RSC Tissue DNA Kit. We used the primers 16Sc-L (5′-GTRGGCCTAAAAGCAGCCAC-3′), and 16Sd-H (5′-CTCCGGTCTGAACTCAGATGACGTAG- 3′) to amplify and sequence the 16S gene [59]. Amplification was done using the following PCR thermal profile: 95 °C for 4 min, followed by 35 cycles of 95 °C for 30 s, 52 °C for 30 s, 72 °C for 70 s, and a final extension phase at 72 °C for 7 min [31]. Amplified DNA products were subsequently visualized on 1.0% agarose gels and sequenced at Genewiz, Frederick, MD. Sequences were assembled, aligned (MUSCLE algorithm), and concatenated in Geneious Pro 5.3 [60] prior to phylogenetic estimation. Collection of specimens used in this study complied with institutional guidelines.

### Phylogenetic analysis

The concatenated sequence matrix was partitioned by gene and the program IQ-TREE v1.6.8 was used to estimate a maximum likelihood (ML) phylogeny [61, 62]. The best-fit substitution model for each partition under the Bayesian information criterion was inferred using ModelFinder [63]. Partitions were allowed to merge by executing the “–m MFP + MERGE” function and the best partition scheme was obtained by examining the top 10% partition-merging schemes using the “–rcluster 10” command [64]. Branch support was assessed with 5000 bootstrap replicates using Ultrafast Bootstrap Approximation (UFB; Hoang et al., [65]). UFB values of 95 and above were considered well supported. We also estimated a Bayesian phylogeny using BEAST v2.5 [66], which was implemented through the CIPRES portal [67]. The best-fit substitution model for each partition was estimated via model averaging using the bModelTest plugin in BEAST [68]. A relaxed log-normal and Yule model was selected for the molecular clock and tree priors with all other priors set to default values. We executed two separate MCMC chains at 50,000,000 generations each and checked for convergence using the program Tracer v1.7 [69]. Sampled trees from both MCMC chains were combined using *logcombiner* and a maximum clade credibility tree was constructed using *treeannotator* with a burn-in of 10%.

### Species delimitation

#### Candidate species

We first grouped samples into populations to form the fundamental units for species delimitation analyses. Based on inferred relationships from the concatenated BEAST phylogeny, populations were defined as monophyletic lineages that also occurred on separate landmasses or biogeographic regions (e.g. PM, Borneo, Java, Sumatra, Sulawesi, Indochina). We then implemented a threshold-based approach to screen for populations with taxonomic incompatibilities (highly diverged conspecific lineages or non-conspecific lineages with low divergences) [5, 45, 57, 70, 71]. To obtain an overview of intra- vs. interspecific genetic variation, pairwise uncorrected p-distances of the 16S mitochondrial gene were calculated using the program PAUP* [72]. We applied thresholds of 3–5%, which have been shown to effectively capture sequence divergences of nascent and fully diverged species in amphibians [45, 46]. Non-conspecific taxon pairs below the thresholds were flagged as candidates for lumping, while conspecific pairs above the thresholds were identified as candidates for splitting.

#### BPP analysis

We used the program BPP v4 [47] to perform species delimitation analyses on the candidate groups. This analysis was based on a single 16S gene, which had the best coverage across all taxa. The A10 analysis (species delimitation using a fixed guide tree) was implemented using relationships derived from phylogenetic analyses as a guide tree. The guide tree was represented by species-level relationships and conspecific individuals were combined under the same nominal identifier. The mapping of individuals to their identifiers was done in the *imap* file supplied to BPP. To explore the sensitivity of the BPP analysis to different prior settings, we tested a series of 10 different prior sets, using α = 3 in the inverse-gamma as a diffuse prior, and α = 21 as an informative prior. The prior mean was then set to vary by two orders of magnitude [73] while the MCMC was set to 100,000 samples with burnin = 10,000 and sample frequency = 5. Convergence was assessed by comparing the consistency of posterior distributions [44, 73, 74]. To accommodate uncertainty in the guide tree, we also performed the A11 analysis (joint species delimitation and species-tree estimation) using the same prior sets. Because these prior settings may not accurately represent the diversification and demographic history of the taxa, we performed separate A10 and A11 analyses using empirical priors calculated from the data. We used α =3 for both θ and τ priors and the corresponding β parameter was adjusted according to the mean (m) estimate of nucleotide diversity (for θ) and node height (for τ) using the equation m = β/(α-1), for α > 2 [73]. The average node height for each candidate group was obtained from the BEAST analysis. Posterior probabilities, pp. ≥ 0.95 were considered highly supported, 0.90 ≤ pp. < 0.95 were considered moderately supported, whereas pp. < 0.9 were considered weakly supported. Finally, we compared posterior distributions generated from priors only, versus those that were generated from empirical data to determine whether the data contained sufficient information for species delimitation and to ensure that the results were not driven by the priors.

#### Heuristic gdi analysis

We implemented the A00 analysis in BPP to generate posterior distributions for the parameters τ and θ using empirical priors. Four separate runs were performed to ensure convergence and converged runs were combined to generate posterior distributions for the MSC parameters that were subsequently used to calculate the *gdi* following the equation: *gdi* = 1 – e^-2τ/ θ^ [43, 44]. Population A is distinguished from population B by using 2τ_AB_/θ_A_, while 2τ_AB_/θ_B_ is used to differentiate population B from population A. Populations are considered distinct species when *gdi* values are > 0.7, while low *gdi* values < 0.2 indicate that populations belong to the same species. Values of 0.2 > *gdi* < 0.7 indicate ambiguous species status [43, 75].

## Additional files

Additional file 1: Maximum likelihood phylogeny derived from 6236 bp comprising three mitochondrial (12S, 16S, CO1) and three nuclear genes (CXCR4, NCX1, RAG-1). Node values denote Ultrafast Bootstrap support values. (PDF 8 kb)

Additional file 2: Bayesian phylogeny derived from 6236 bp comprising three mitochondrial (12S, 16S, CO1) and three nuclear genes (CXCR4, NCX1, RAG-1). Node values denote Bayesian posterior probabilities. (PDF 7 kb)

Additional file 3: Posterior distributions of tau generated from priors versus empirical data. (PDF 3012 kb)

Additional file 4: Molecular samples used in this study and their associated GenBank accession number. The asterisk (*) denotes newly sequenced samples for this study. (XLSX 11 kb)

Additional file 5: Pairwise uncorrected p-distances of the 16S gene. (CSV 6 kb)

Additional file 6: Results of the BPP A11 analysis. (ZIP 216 kb)

## Abbreviations

BPP: Bayesian Phylogenetics and Phylogeography
gdi: Genealogical divergence index
MCMC: Markov Chain Monte Carlo
ML: Maximum likelihood
MSC: Multispecies Coalescent
PM: Peninsular Malaysia
PP: Posterior probability
rjMCMC: Reversible-jump Markov Chain Monte Carlo
