# Supplementary figures and images for: To split or not to split? Multilocus phylogeny and molecular species delimitation of southeast Asian toads (family: Bufonidae)
Source: BMC Evol Biol. 2019 Apr 25;19:95. doi: 10.1186/s12862-019-1422-3 (PMC6485082; doi:10.1186/s12862-019-1422-3)

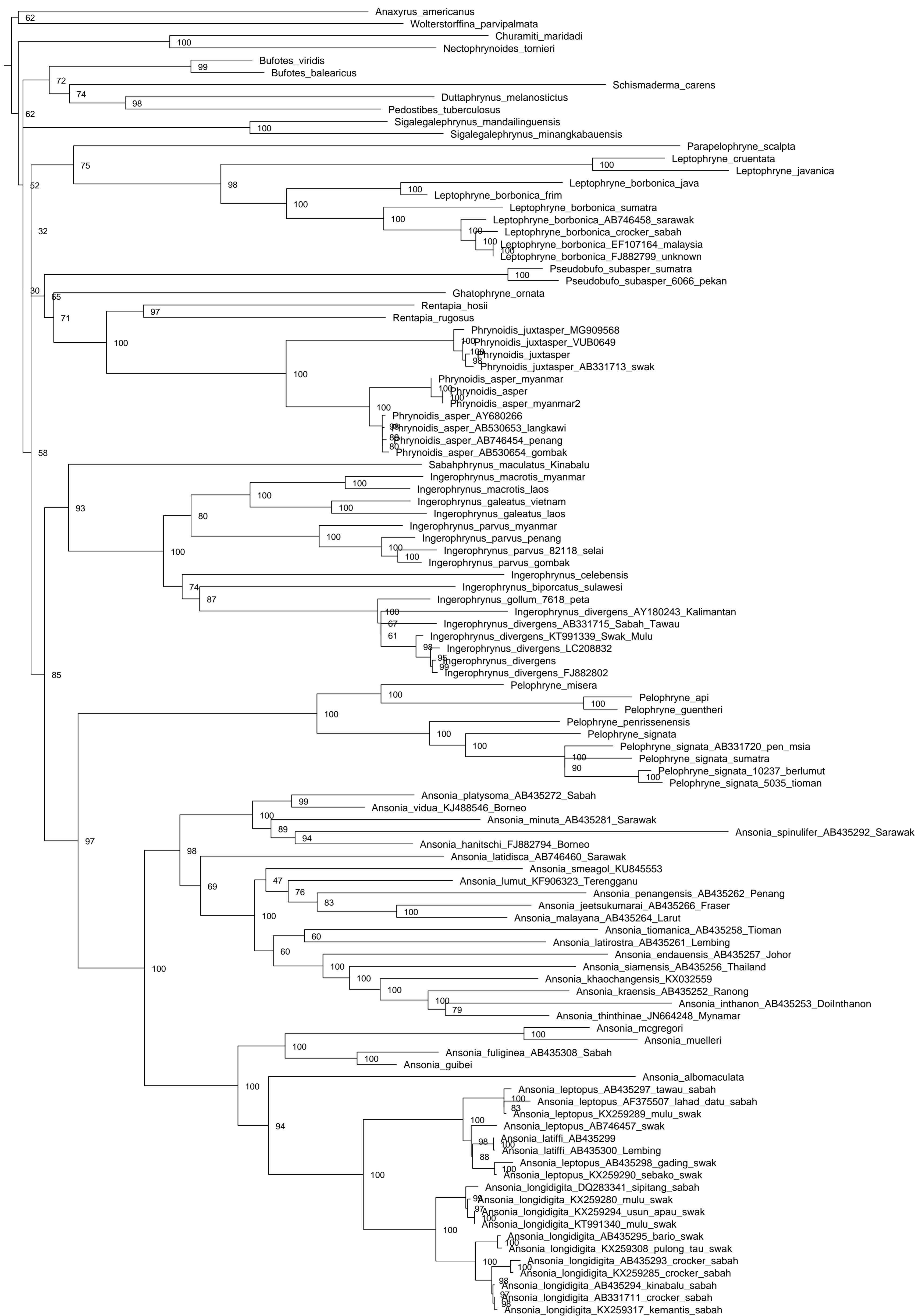

0.03

Supplement: Supplementary file 1 — Maximum likelihood phylogeny derived from 6236 bp comprising three mitochondrial (12S, 16S, CO1) and three nuclear genes (CXCR4, NCX1, RAG-1). Node values denote Ultrafast Bootstrap support values. (PDF 8 kb) [file 12862_2019_1422_MOESM1_ESM.pdf]

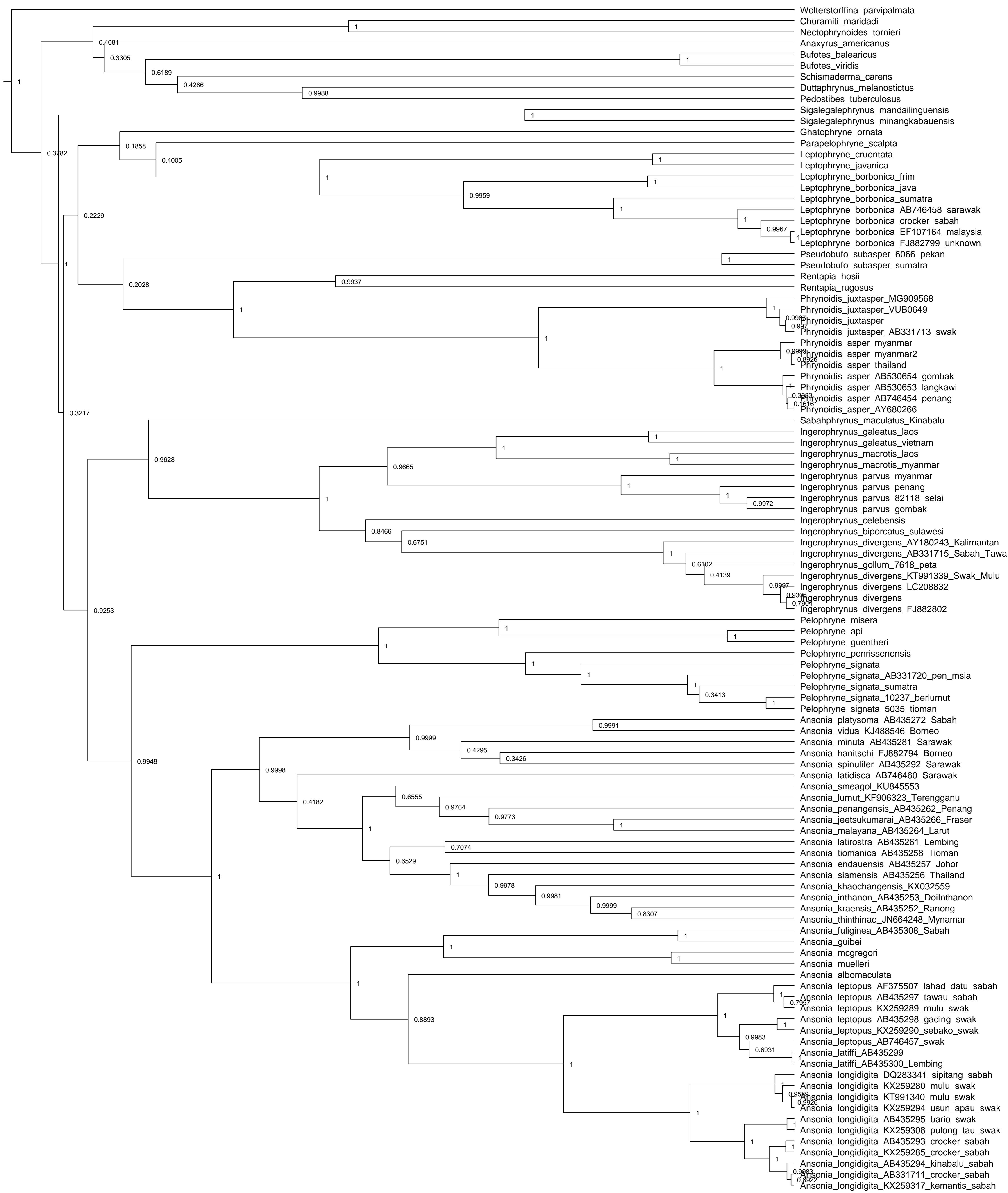

0.02

Supplement: Supplementary file 2 — Bayesian phylogeny derived from 6236 bp comprising three mitochondrial (12S, 16S, CO1) and three nuclear genes (CXCR4, NCX1, RAG-1). Node values denote Bayesian posterior probabilities. (PDF 7 kb) [file 12862_2019_1422_MOESM2_ESM.pdf]

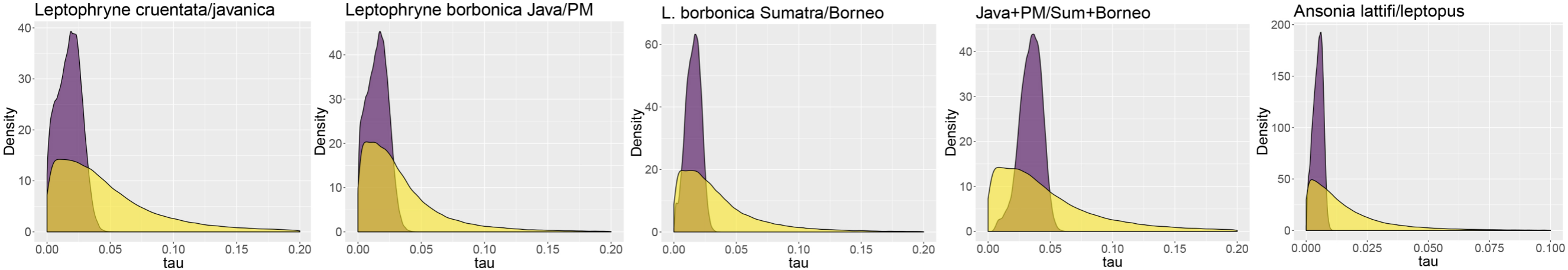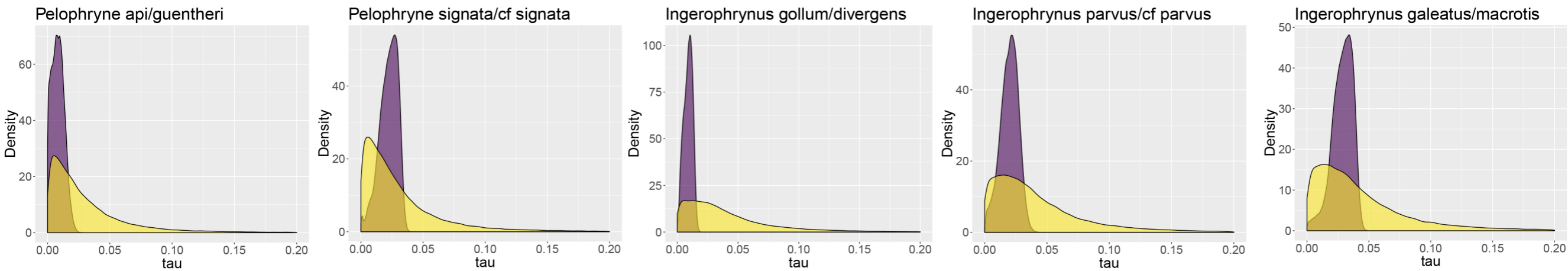

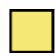 Prior only

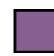 Empirical

Supplement: Supplementary file 3 — Posterior distributions of tau generated from priors versus empirical data. (PDF 3012 kb) [file 12862_2019_1422_MOESM3_ESM.pdf]
